# Specific effects of distinct types of adverse childhood experiences on the co-occurrence of non-suicidal self-injury and suicidal behaviors among Chinese adolescents: a latent class analysis

**DOI:** 10.3389/fpsyt.2025.1698537

**Published:** 2026-01-12

**Authors:** Zhouyan Wang, Wei Chen, Siwei Yang, Gen Chen, Xiaoke Wan, Xia Li, Chang Peng, Hong Wang

**Affiliations:** 1 College of Public Health, Chongqing Medical University, Chongqing, China; 2 Research Center for Medicine and Social Development, Chongqing Medical University, Chongqing, China; 3 Bishan Hospital of Chongqing Medical University/Bishan Hospital of Chongqing, Chongqing, China; 4 Hechuan District Center for Disease Control and Prevention, Chongqing, China

**Keywords:** adolescent, adverse childhood experiences, latent class analysis, non-suicidal self-injury, suicidal behaviors

## Abstract

**Background:**

Adverse childhood experiences (ACEs) are well-established risk factors for both non-suicidal self-injury (NSSI) and suicidal behaviors (SBs). Actually, NSSI and SBs often co-occur among adolescents. This study aims to examine the specific effects of ACE types on the co-occurrence of NSSI and three stages of SBs in Chinese adolescents, as well as potential gender differences.

**Methods:**

A cross-sectional study was conducted in Chongqing, China. From April to May 2024. A total of 5,143 junior high school students were recruited through stratified cluster sampling. Information was collected through a self-reported questionnaire. Latent class analysis (LCA) was used to identify the co-occurrence patterns of NSSI and SBs. Multinomial logistic regression was employed to examine the independent associations between ACE types and these co-occurrence patterns. Gender differences were assessed using relative odds ratios (*ROR*).

**Results:**

The prevalence of different ACE types ranged from 2.5% to 23.0%, with 46.4% of participants reporting one or more ACEs. Three co-occurrence patterns of NSSI and SBs were identified: low risk class, suicidal ideation class, and suicide attempts class. Physical abuse (odds ratios [*OR*] = 1.50, 95% confidence intervals [*CI*] 1.05 to 2.15) was associated with suicidal ideation class, while emotional abuse (*OR* = 1.88, 95% *CI* 1.18 to 3.02) and neglect (*OR* = 1.45, 95% *CI* 1.01 to 2.09) were associated with suicide attempts class. A gender difference was found in the association between neglect and suicide attempts class (*ROR* = 2.34, 95% *CI* 1.14 to 4.83).

**Conclusions:**

Suicide attempts (SA) and suicidal ideation (SI) tend to co-occur with NSSI among adolescents. Distinct ACE types have unique effects on this co-occurrence, with gender differences. These findings emphasize the importance of considering NSSI and SBs together, and it is more promising to develop tailored anti-ACEs interventions.

## 1 Introduction

Non-suicidal self-injury (NSSI) and suicide are major public health concerns worldwide, with the greatest burden of both occurring in low-and middle-income countries (LMICs) (1). NSSI refers to direct, deliberate self-inflicted destruction of body tissues without suicidal intent or specific purposes (2, 3). It was estimated that 14.6 million individuals are affected by NSSI each year (4). In China, the 12-month prevalence of NSSI among middle school students is 30.4% (5). Suicide, defined as a fatal self-injurious act with some evidence of intent to die at least (6, 7), accounts for more than 720,000 deaths annually worldwide (8). Suicide is typically conceptualized as a spectrum of suicidal behavior (SBs), including suicidal ideation (SI), suicide plans (SP), and suicide attempts (SA) (9, 10). Among Chinese children and adolescents, the detection rates for SI, SP, and SA are reported as 15.4%, 6.4% and 3.5%, respectively (11).

Although NSSI and SBs are distinct forms of behavior, an increasing number of studies have found that they often co-occur in adolescent populations (12–14). For example, one study reported a 6.6% co-occurrence rate of NSSI and SBs among adolescents (14). More specifically, some studies have documented co-occurrence rates of 4.1% for NSSI and SI, 3.4% for NSSI and SP, 10.0% for NSSI and SA (14–16). While numerous studies have investigated the overlap between NSSI and SBs, existing research is predominantly variable-based. This approach can only explore the co-occurrence of NSSI with a single stage of SBs, failing to provide a more comprehensive examination. With the advent of statistical methods, latent class analysis (LCA) has emerged as a novel approach to explore patterns of behavioral co-occurrence (17, 18). Unlike the traditional variable-centered approach, LCA focuses on the relationship among individuals (19), assuming that heterogeneous individuals within a population can be grouped into smaller, relatively homogeneous subgroups that display similar patterns of behaviors or trait expressions (20). This approach contributes to the development of tailored interventions, both clinically and practically (21, 22). Several studies have employed LCA to explore the co-occurrence of NSSI and SBs (NSSI + SBs). For instance, a study conducted among gun owners in the US identified five patterns of NSSI and SBs (23). Dhingra et al.’s study categorized NSSI and SBs among university students into three subgroups (21). However, these studies have primarily focused on adults or special groups. There is a need to investigate the co-occurrence patterns in adolescents to better understand the underlying connections within adolescent self-injurious behaviors.

A study has shown that individuals are at a higher risk of suicide death when NSSI and SBs occur concurrently (24). Given the prevalence of co-occurrence patterns and the high-risk characteristics of individuals affected, identifying early modifiable risk factors is of crucial importance. Among these factors, adverse childhood experiences (ACEs), referring to some of the most intensive and frequently occurring sources of stress that children may suffer early in life (25), have emerged as a critical area of focus in related research. ACEs typically include abuse, neglect, and household dysfunction, et al. A meta-analysis reported that 72.5% of adolescents suffered at least one ACE globally (26). It was demonstrated that ACEs are widely associated with physical, psychological, and behavioral health outcomes (27, 28). The associations of ACEs with adolescent NSSI and SBs have also been repeatedly validated (29–34).

Limited studies explored the effects of ACEs on NSSI + SBs. A case-control study conducted among opioid dependent individuals in Australia showed that ACEs increase the risk of NSSI + SBs (35). One school-based survey involving 14,500 Chinese adolescents found that ACEs are an influencing factor for NSSI + SBs (36). Hamza et al. proposed an integrated model by synthesizing various theories (37). On one hand, the observed relationship between NSSI and SBs may be spurious, with shared risk factors such as ACEs and lack of family support driving their co-occurrence (38). On the other hand, NSSI may directly predict SBs or indirectly predict SBs mediated by the acquired capability for suicide (39). Despite progress, critical limitations remain in existing research. First, current studies focus primarily on traditional core types of ACEs, such as abuse and neglect. As the scope of ACE research expands, it is important to include a wider range of ACE items to evaluate their relationship with NSSI + SBs comprehensively (40, 41). Second, there is a lack of exploration into the exact effects of different ACE types on the co-occurrence. When examining these effects, the interrelatedness of ACEs and their tendency to co-occur are often overlooked, which may lead to an overestimation of the contribution of any single ACE type (42, 43). In other words, existing studies fail to identify which types of ACEs are the most decisive risk factors, which could inform targeted prevention efforts. Furthermore, no prior work has explored gender differences in the associations between ACEs and NSSI + SBs among adolescents. Current research suggests that gender may be an important influencing factor for the co-occurrence (13, 15, 44). The association between gender and NSSI + SBs has been examined, but inconsistent results have emerged from available studies. While some studies have shown that girls have a higher prevalence of NSSI + SBs than boys (36, 45, 46), other studies report the opposite (47). In terms of co-occurrence patterns, the findings are similarly inconsistent. One study suggested that males are more likely to fall into the high-risk group (20), while another study reached the opposite conclusion (21).

In summary, the existing literature has several limitations that need to be addressed. First, few studies have specifically investigated the co-occurrence patterns of NSSI and SBs in adolescents. Second, prior research has not examined the unique contributions of different ACE types to NSSI + SBs, particularly in the context of including a broader range of ACE items. Third, the role of gender in the relationship between ACEs and NSSI + SBs remains unclear. Therefore, the current study aims to achieve three objectives: first, to identify the co-occurrence patterns of NSSI and SBs among Chinese adolescents via LCA; second, to examine how different types of ACEs are associated with these co-occurrence patterns; third, to explore whether gender differences exist in the associations between ACEs and co-occurrence patterns.

## 2 Materials and methods

### 2.1 Procedures and participants

The study employed a cross-sectional design and field investigations conducted between April and May 2024. Participants were recruited through multi-stage cluster sampling from middle schools in Chongqing, a city located in the central-western region of China. Initially, two districts (A and B) representing the city’s main urban and county areas were selected based on an economic status-based stratified sampling frame. Next, two middle schools (one urban and one rural) were selected from each district, totaling eight schools. Then, four to six classes from each grade (7th to 9th) were selected with the assistance of school administrators. Finally, all students present in the selected class were invited to participate in the study voluntarily.

This study was approved by the medical research ethical committee of Chongqing Medical University (Grant No. 2023028). All students voluntarily took part in the study. Prior to the field investigation, students were fully informed about the study and provided their consent. In addition, students were made aware that they could access free counseling for psychological distress during the survey, and those in need could also seek online support from us afterward.

Self-reported questionnaires were distributed to 5,143 students from the selected junior high schools. A total of 5,040 questionnaires were completed without apparent logical errors (e.g., selecting the same option for all questions), and with no more than 15% missing items. The valid response rate was 98.00% (5,040/5,143).

### 2.2 Measurements

#### 2.2.1 ACEs

In this study, ACEs were defined as childhood maltreatment and household dysfunction (48). Childhood maltreatment was measured via the Revised Version of Childhood Trauma Questionnaire-Short Form (CTQ-SF-RV), revised by Peng et al. (49). The revised scale has demonstrated good reliability and validity in Chinese adolescents. It contains 27 items (24 clinical items and 3 validity items) across four dimensions: physical abuse, emotional abuse, sexual abuse, and neglect. Items are rated on Likert 5 (1 = never, 2 = rarely, 3 = sometimes, 4 = often, 5 = always). Seven items (2, 5, 7, 13, 19, 26, 28) are reverse-scored. The total scores of each dimension were categorized into dichotomous variables, with the following positive cutoff values: physical abuse > 8, emotional abuse > 11, sexual abuse > 7, and neglect > 26. The Cronbach’s α for the CTQ-SF-RV in this study was 0.733, with the four dimensions ranging from 0.715 to 0.836.

The household dysfunction items were adapted from Felitti et al. and assessed based on the following experiences (48): (1) household substance abuse, (2) parental separation/divorce, (3) witnessed mother abused, (4) household mental illness, (5) household incarceration. Each item offers two responses: “yes” or “no.” Respondents were considered “exposed to the item” if they answered “yes.” This method has been used to measure the experiences of household dysfunction among Chinese high school students and has demonstrated good applicability and reliability (50, 51).

#### 2.2.2 NSSI

NSSI was measured via the Adolescent Non-suicidal Self-injury Assessment Questionnaire (ANSAQ) developed by Wan et al. (52), which assesses NSSI over the past 12 months. The questionnaire includes 12 types of self-injury behaviors, such as “suffocation” and “scratching.” Responses range from 1 to 4 (1 = 0 times, 2 = 1 time, 3 = 2 ~ 4 times, 4 = ≥ 5 times). According to DSM-5 standards, participants with a cumulative frequency of ≥ 5 were classified as engaging in NSSI (53). The Cronbach’s α for the ANSAQ in the current study was 0.901.

#### 2.2.3 SBs

In the current study, SBs incorporate three stages: SI, SP, and SA. Three questions were used to measure students’ SBs over the past 12 months: (1) Have you seriously thought about suicide? (SI); (2) Have you made a suicide plan? (SP); (3) Have you taken steps to attempt suicide? (SA). Respondents were identified as having the corresponding SBs if they answered “yes” to any of the questions. Based on the detection of SBs, participants were categorized into four groups: none (no SBs), SI (only SI, without SP and SA), SP (SI and SP, without SA), and SA (SI, SP, and SA) (10, 54).

#### 2.2.4 Demographic and confounding factors

Demographic variables included grade (7th, 8th, or 9th), gender (male or female), only child status (yes or no), boarding status (yes or no), residence (urban or rural), paternal education level (primary school or less, high school, college or above), maternal education level (primary school or less, high school, college or above), family economic status (poor, average, or good), academic performance (bad, moderate, or good), learning burden (light, medium, or heavy), and number of intimate friends (none, 1 ~ 2, 3 ~ 5, 6 or more).

Depression was measured via the Center for Epidemiologic Studies-Depression Scale (CES-D) developed by Radloff (55). It has been proven reliable and valid in the Chinese adolescent population (56). The CES-D consists of 20 items that screen for depression symptoms experienced over the past week. Responses range from 0 to 3 for each item (0 = rarely, 1 = some of the time, 2 = often, 3 = most of the time). Four items (4, 8, 12, and 16) are reverse-scored. The total score ranges from 0 to 60, with higher scores indicating more severe depression symptoms (57). A total score of 16 or higher was defined as clinically relevant depressive symptoms (58). The Cronbach’s α of CES-D in this study was 0.879.

### 2.3 Statistical analyses

All analyses were performed using Mplus 8.3 and SPSS 27.0. The data were cleaned before analysis, and a small number of non-critical massing values were reasonably imputed using simple imputation methods. The formal statistical analyses included the following steps: First, LCA was conducted to examine the co-occurrence of NSSI and SBs, and to classify participants with similar patterns. A series of models were fitted sequentially, estimating 1 to 5 classes. Model fit indices include Akaike Information Criterion (AIC), Bayesian Information Criterion (BIC), sample-size adjusted BIC (aBIC), entropy, Lo-Mendell-Rubin adjusted likelihood ratio test (LMR), and Bootstrap Likelihood Ratio Test (BLRT) (59). Smaller values of AIC, BIC, and aBIC indicate better model fit. Entropy values range from 0 to 1, with higher values suggesting better model separation. When entropy exceeds 0.8, the classification accuracy is above 90.0% (60). Significant *p*-value*s* for LMR and BLRT indicate that the k-class model is superior to the k-1 class model. The optimal model selection was based on smaller values of AIC, BIC, and aBIC, higher values of entropy, significance of LMR and BLRT, and the interpretability of the model. After identifying the best-fitting latent class solution, a single categorical variable was created to represent the classes (61). Second, the distribution of categorical variables was summarized by [n (%)], and continuous variables were described by ( $\bar{x}\pm s$). The chi-square test was used to examine the associations between two categorical variables. Third, a multinomial logistic regression model was applied to assess the independent associations between different types of ACEs and the co-occurrence patterns of NSSI and SBs, adjusting for the other 8 types of ACEs. Associations were reported as odds ratios (*OR*) with 95% confidence intervals (95% *CI*). Finally, the gender differences in these associations were tested via interaction terms and presented as relative odds ratios (*ROR*) (62). The significance level was set at *p* < 0.05 (two-tailed).

## 3 Results

### 3.1 Co-occurrence patterns of NSSI and SBs

LCA models with one to five classes were tested to identify the co-occurrence patterns of NSSI and SBs. The 3-class model was determined to be optimal based on lower AIC, BIC, and aBIC values, higher entropy, and the significance of LMR and BLRT. Detailed model fit indices are provided in **Table 1**.

**Table 1:** Fit indices for latent class models of NSSI and SBs.

Model	AIC	BIC	aBIC	Entropy	LMR	BLRT	n (%)				
					*p*	*p*	1	2	3	4	5
1	15973.721	15999.821	15987.111	1.000			5,040 (100.00)				
2	12425.173	12483.899	12455.300	0.901	< 0.001	< 0.001	4,186 (83.06)	854 (16.94)			
3	12173.125	12264.478	12219.990	0.890	< 0.001	< 0.001	3,653 (72.48)	1,166 (23.14)	221 (4.39)		
4	12183.125	12307.103	12246.728	0.767	0.500	1.000	0 (0.00)	3,653 (72.48)	221 (4.39)	1,166 (23.14)	
5	12193.125	12349.729	12273.465	0.640	0.501	1.000	0 (0.00)	3,415 (67.76)	412 (8.18)	975 (19.35)	238 (4.72)

AIC, Akaike Information Criterion; BIC, Bayesian Information Criterion; aBIC, sample-size adjusted BIC; LMR, Lo-Mendell-Rubin adjusted likelihood ratio test; BLRT, Bootstrap Likelihood Ratio Test.

**Figure 1:**
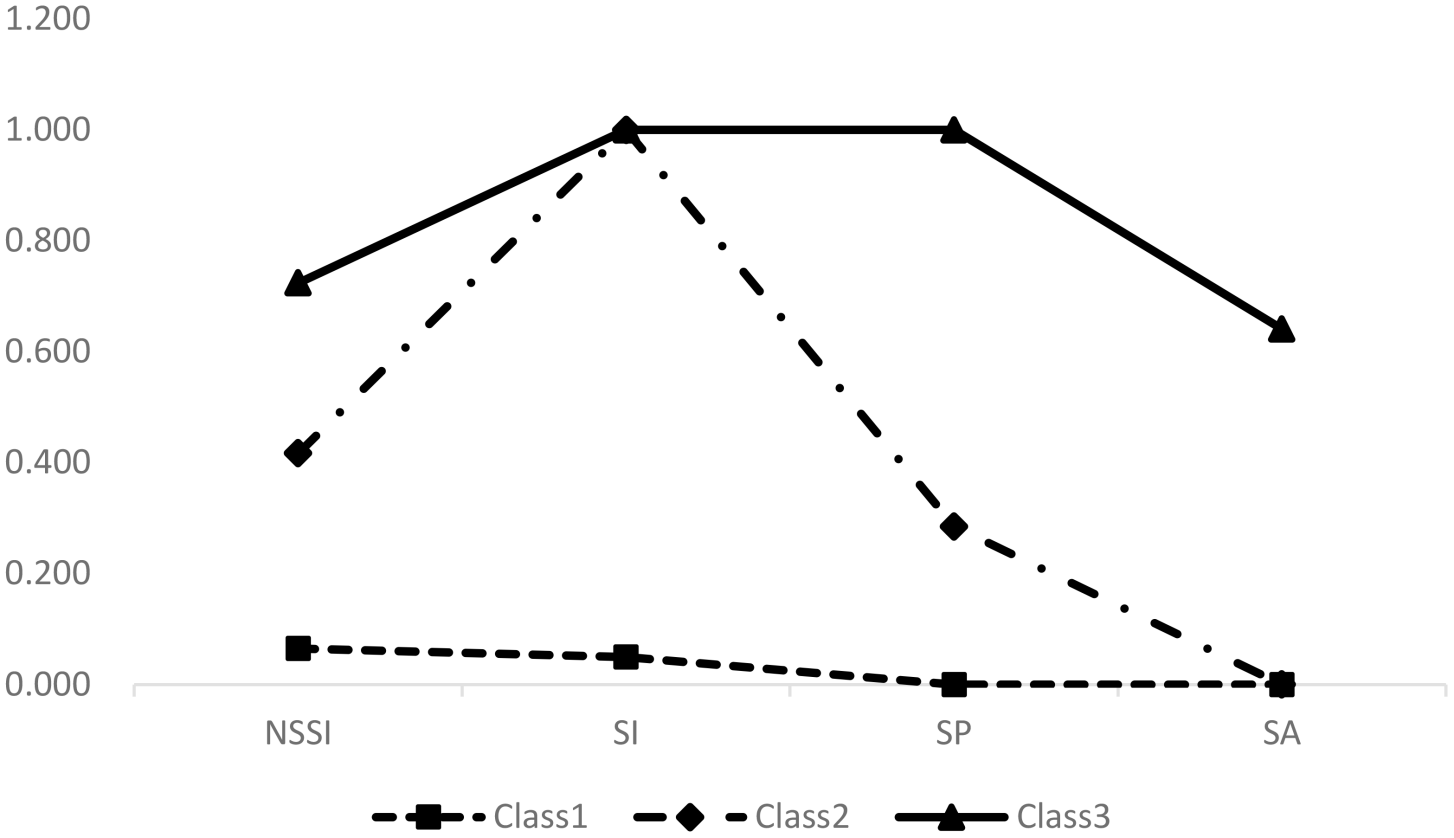
Plots of three latent classes for the NSSI and SBs.

**Figure 1** illustrates the 3-class model and item-response probabilities for the four behaviors in each class. Specific values are available in **Supplementary Material (Table S3)**. Class 1 was identified as low risk class (3,653 participants, 72.48%), consisting of individuals with low probabilities of engaging in NSSI and the three forms of SBs. Class 2 was recognized as suicidal ideation class (1,166 participants, 23.14%), comprising participants with a high probability of SI and moderate probabilities of NSSI. Class 3 was labeled as suicide attempts class (221 participants, 4.39%), covering students with high probabilities of engaging in all four behaviors, particularly SP and SA. In both subgroups (males vs. females), the patterns of NSSI and SBs were similar, so we conducted LCA on the entire sample for the analysis. Further details are provided in the **Supplementary Material (Table S1–S2, Table S4–S5, Figure S1–S2)**.

### 3.2 Sample characteristics

The mean age of participants was (13.8 ± 1.0) years. Among the 5,040 participants, more than half (51.3%) were female, and 48.7% were male. 46.4% of participants reported experienced at least one ACE prior to the survey. Specifically, 25.9% reported only one ACE, 16.2% reported two or three ACEs, and 4.3% reported four or more ACEs. The prevalence rates of NSSI, SI, SP, and SA were 17.0%, 27.5%, 11.7%, and 4.4%, respectively. The proportions of suicidal ideation class (27.8% vs. 18.3%) and suicide attempts class (5.7% vs. 3.0%) were higher among females than males, with these differences being statistically significant (*ps* all < 0.05). More detailed participant characteristics are provided in **Table 2**.

**Table 2:** General characteristics of three classes of NSSI and SBs among the study population (N = 5,040).

Variables	Total		Low risk class		Suicidal ideation class		Suicide attempts class		*χ^2^*
	n	% (95% *CI*)	n	% (95% *CI*)	n	% (95% *CI*)	n	% (95% *CI*)	
Grade									64.895 ^***^
7th	1,544	30.6 (29.4 ~ 31.9)	1,018	65.9 (63.6 ~ 68.3)	444	28.8 (26.5 ~ 31.0)	82	5.3 (4.2 ~ 6.4)	
8th	1,781	35.3 (34.0 ~3 6.7)	1,288	72.3 (70.2 ~ 74.4)	414	23.2 (21.3 ~ 25.2)	79	4.4 (3.5 ~ 5.4)	
9th	1,715	34.0 (32.7 ~ 35.3)	1,347	78.5 (76.6 ~ 80.5)	308	18.0 (16.1 ~ 19.8)	60	3.5 (2.6 ~ 4.4)	
Gender									97.003 ^***^
Male	2,454	48.7 (47.3 ~ 50.1)	1,933	78.8 (77.2 ~ 80.4)	448	18.3 (16.7 ~ 19.8)	73	3.0 (2.3 ~ 3.6)	
Female	2,586	51.3 (50.0 ~ 52.7)	1,720	66.5 (64.7 ~ 68.3)	718	27.8 (26.0 ~ 29.5)	148	5.7 (4.8 ~ 6.6)	
Only child status									0.583
Yes	964	19.1 (18.0 ~ 20.2)	690	71.6 (68.7 ~ 74.4)	232	24.1 (21.4 ~ 26.8)	42	4.4 (3.1~ 5. 6)	
No	4,076	80.9 (79.8 ~ 82.0)	2,963	72.7 (71.3 ~ 74.1)	934	22.9 (21.6 ~ 24.2)	179	4.4 (3.8 ~ 5.0)	
Boarding status									6.158 ^*^
Yes	2,294	45.5 (44.1 ~ 46.9)	1,625	70.8 (69.0 ~ 72.7)	567	24.7 (23.0 ~ 26.5)	102	4.4 (3.6 ~ 5.3)	
No	2,746	54.5 (53.1 ~ 55.9)	2,028	73.9 (72.2 ~ 75.5)	599	21.8 (20.3 ~ 23.4)	119	4.3 (3.6 ~ 5.1)	
Residence									4.084
Rural	2,611	51.8 (50.4 ~ 53.2)	1,886	72.2 (70.5 ~ 74.0)	596	22.8 (21.2 ~ 24.4)	129	4.9 (4.1 ~ 5.8)	
Urban	2,429	48.2 (46.8 ~ 49.6)	1,767	72.7 (71.0~74.5)	570	23.5 (21.8 ~ 25.2)	92	3.8 (3.0 ~ 4.5)	
Paternal education level									7.166
Primary school or less	730	14.5 (13.5 ~ 15.5)	500	68.5 (65.1 ~ 71.9)	191	26.2 (23.0~29.4)	39	5.3 (3.7 ~ 7.0)	
High school	3,875	76.9 (75.7 ~ 78.0)	2,833	73.1 (71.7 ~ 74.5)	877	22.6 (21.3 ~ 24.0)	165	4.3 (3.6 ~ 4.9)	
College or more	435	8.6 (7.9 ~ 9.4)	320	73.6 (69.4 ~ 77.7)	98	22.5 (18.6 ~ 26.5)	17	3.9 (2.1 ~ 5.7)	
Maternal education level									2.716
Primary school or less	821	16.3 (15.3 ~ 17.3)	582	70.9 (67.8 ~ 74.0)	199	24.2 (21.3 ~ 27.2)	40	4.9 (3.4 ~ 6.3)	
High school	3,869	76.8 (75.6 ~ 77.9)	2,820	72.9 (71.5 ~ 74.3)	880	22.7 (21.4 ~ 24.1)	169	4.4 (3.7 ~ 5.0)	
College or more	350	6.9 (6.2 ~ 7.6)	251	71.7 (67.0 ~ 76.5)	87	24.9 (20.3 ~ 29.4)	12	3.4 (1.5 ~ 5.3)	
Family economic status									77.692 ^***^
Poor	549	10.9 (10.0 ~ 11.8)	320	58.3 (54.2 ~ 62.4)	182	33.2 (29.2 ~ 37.1)	47	8.6 (6.2 ~ 10.9)	
Average	3,376	67.0 (65.7 ~ 68.3)	2,471	73.2 (71.7 ~ 74.7)	777	23.0 (21.6 ~ 24.4)	128	3.8 (3.1 ~ 4.4)	
Good	1,115	22.1 (21.0 ~ 23.3)	862	77.3 (74.8 ~ 79.8)	207	18.6 (16.3 ~ 20.9)	46	4.1 (3.0 ~ 5.3)	
Academic performance									75.968 ^***^
Bad	1,847	36.6 (35.3 ~ 38.0)	1,220	66.1 (63.9 ~ 68.2)	503	27.2 (25.2 ~ 29.3)	124)	6.7 (5.6 ~ 7.9)	
Moderate	1,772	35.2 (33.8 ~ 36.5)	1,348	76.1 (74.1 ~ 78.1)	362	20.4 (18.5 ~ 22.3)	62	3.5 (2.6 ~ 4.4)	
Good	1,421	28.2 (27.0 ~ 29.4)	1,085	76.4 (74.1 ~ 78.6)	301	21.2 (19.1 ~ 23.3)	35	2.5 (1.7 ~ 3.3)	
Learning burden									250.324 ^***^
Light	611	12.1 (11.2 ~ 13.0)	513	84.0 (81.0 ~ 86.9)	89	14.6 (11.8 ~ 17.4)	9	1.5 (0.5 ~ 2.4)	
Medium	2,786	55.3 (53.9 ~ 56.7)	2,177	78.1 (76.6 ~ 79.7)	521	18.7 (17.3 ~ 20.1)	88	3.2 (2.5 ~ 3.8)	
Heavy	1,643	32.6 (31.3 ~ 33.9)	963	58.6 (56.2 ~ 61.0)	556	33.8 (31.6 ~ 36.1)	124	7.5 (6.3 ~ 8.8)	
Number of intimate friends									147.429 ^***^
None	125	2.5 (2.1 ~ 2.9)	53	42.4 (33.6 ~ 51.2)	52	41.6 (32.8 ~ 50.4)	20	16.0 (9.5 ~ 22.5)	
1 ~ 2	1,418	28.1 (26.9 ~ 29.4)	926	65.3 (62.8 ~ 67.8)	406	28.6 (26.3 ~ 31.0)	86	6.1 (4.8 ~ 7.3)	
3 ~ 5	2,124	42.1 (40.8 ~ 43.5)	1,590	74.9 (73.0 ~ 76.7)	462	21.8 (20.0 ~ 23.5)	72	3.4 (2.6 ~ 4.2)	
6 or more	1,373	27.2 (26.0 ~ 28.5)	1,084	79.0 (76.8 ~ 81.1)	246	17.9 (15.9 ~ 19.9)	43	3.1 (2.2 ~ 4.1)	
Number of ACEs									520.980 ^***^
None	2,702	53.6 (52.2 ~ 55.0)	2,203	81.5 (80.1 ~ 83.0)	450	16.7 (15.2 ~ 18.1)	49	1.8 (1.3 ~ 2.3)	
1	1,306	25.9 (24.7 ~ 27.1)	940	72.0 (69.5 ~ 74.4)	320	24.5 (22.2 ~ 26.8)	46	3.5 (2.5 ~ 4.5)	
2 ~ 3	815	16.2 (15.2 ~ 17.2)	430	52.8 (49.3 ~ 56.2)	308	37.8 (34.5 ~ 41.1)	77	9.4 (7.4 ~ 11.5)	
4 or more	217	4.3 (3.7 ~ 4.9)	80	36.9 (30.4 ~ 43.3)	88	40.6 (34.0 ~ 47.1)	49	22.6 (17.0 ~ 28.2)	
Depression symptom									1186.423 ^***^
Yes	1748	34.7 (33.4 ~ 36.0)	751	43.0 (40.6 ~ 45.3)	811	46.4 (44.1 ~ 48.7)	186	10.6 (9.2 ~ 12.1)	
No	3292	65.3 (64.0 ~ 66.6)	2902	88.2 (87.0 ~ 89.3)	355	10.8 (9.7 ~ 11.8)	35	1.1 (0.7 ~ 1.4)	

*p < 0.05, **p < 0.01, ***p < 0.001. Bold values indicate statistically significant results.

### 3.3 The prevalence of ACE types in males vs. females

The three most prevalent types of ACEs were parental separation/divorce (23.0%), neglect (15.1%), and witnessed mother abused (10.2%). In contrast, the three least prevalent types were household incarceration (2.5%), household mental illness (3.2%), and sexual abuse (4.1%). Except for neglect and household incarceration, the prevalence of other ACE types differed significantly between genders (*ps* all< 0.05). Additional details are provided in **Table 3**.

**Table 3:** Prevalence of ACE types between males and females.

Variables	Total		Males		Females		*χ^2^*
	n	% (95% CI)	n	% (95% CI)	n	% (95% CI)	0.882
ACEs							
Yes	2,338	46.4 (45.0 ~ 47.8)	1155	47.1 (45.1 ~ 49.0)	1183	45.7 (43.8 ~ 47.7)	
No	2,702	53.6 (52.2 ~ 55.0)	1299	52.9 (51.0 ~ 54.9)	1403	54.3 (52.3 ~ 56.2)	
Physical abuse							14.683 ^***^
Yes	268	5.3 (4. 7~ 5.9)	161	6.6 (5.6 ~ 7.5)	107	4.1 (3.4 ~ 4.9)	
No	4,772	94.7 (94.1 ~ 95.3)	2,293	93.4 (92.5 ~ 94.4)	2,479	95.9 (95.1 ~ 96.6)	
Emotional abuse							10.268 ^**^
Yes	331	6.6 (5.9 ~ 7.3)	133	5.4 (4.5 ~ 6.3)	198	7.7 (6.6 ~ 8.7)	
No	4,709	93.4 (92.7 ~ 94.1)	2,321	94.6 (93.7 ~ 95.5)	2,388	92.3 (91.3 ~ 93.4)	
Sexual abuse							14.335 ^**^
Yes	208	4.1 (3.6 ~ 4.7)	128	5.2 (4.3 ~ 6.1)	80	3.1 (2.4 ~ 3.8)	
No	4,832	95.9 (95.3 ~ 96.4)	2,326	94.8 (93.9 ~ 95.7)	2,506	96.9 (96.2 ~ 97.6)	
Neglect							1.891
Yes	763	15.1 (14.1 ~ 16.1)	389	15.9 (14.4 ~ 17.3)	374	14.5 (13.1 ~ 15.8)	
No	4,277	84.9 (83.9 ~ 85.9)	2,065	84.1 (82.7 ~ 85.6)	2,212	85.5 (84.2 ~ 86.9)	
Household substance abuse							4.790 ^*^
Yes	338	6.7 (6.0 ~ 7.4)	184	7.5 (6.5 ~ 8.5)	154	6.0 (5.0 ~ 6.9)	
No	4,702	93.3 (92.6 ~ 94.0)	2,270	92.5 (91.5 ~ 93.5)	2,432	94.0 (93.1 ~ 95.0)	
Parental separation/divorce							4.699 ^*^
Yes	1,157	23.0 (21.8 ~ 24.1)	531	21.6 (20.0 ~ 23.3)	626	24.2 (22.6 ~ 25.9)	
No	3,883	77.0 (75.9 ~ 78.2)	1,923	78.4 (76.7 ~ 80.0)	1,960	75.8 (74.1 ~ 77.4)	
Witnessed mother abused							6.448 ^*^
Yes	514	10.2 (9.4 ~ 11.0)	223	9.1 (7.9 ~ 10.2)	291	11.3 (10.0 ~ 12.5)	
No	4,526	89.8 (89.0 ~ 90.6)	2,231	90.9 (89.8 ~ 92.1)	2,295	88.7 (87.5 ~ 90.0)	
Household mental illness							8.283 ^**^
Yes	160	3.2 (2.7 ~ 3.7)	60	2.4 (1.8 ~ 3.1)	100	3.9 (3.1 ~ 4.6)	
No	4,880	96.8 (96.3 ~ 97.3)	2,394	97.6 (96.9 ~ 98.2)	2,486	96.1 (95.4 ~ 96.9)	
Household incarceration							1.891
Yes	128	2.5 (2.1 ~ 3.0)	70	2.9 (2.2 ~ 3.5)	58	2.2 (1.7 ~ 2.8)	
No	4,912	97.5 (97.0 ~ 97.9)	2,384	97.1 (96.5 ~ 97.8)	2,528	97.8 (97.2 ~ 98.3)	

^*^p < 0.05, ^**^p < 0.01, ^***^p < 0.00. Bold values indicate statistically significant results.

### 3.4 Associations between ACE types and the co-occurrence patterns

To evaluate the independent association between each ACE type and the co-occurrence patterns, all ACE types were included simultaneously in a multinomial regression model, controlling for demographic variables that showed statistical significance and depression scores. Multicollinearity diagnostics conducted prior to the formal analysis revealed no issues (tolerance: 0.804 ~ 0.943, VIF: 1.060 ~ 1.244). Logistic regression result indicated that physical abuse was positively associated with the suicidal ideation class (*OR* = 1.50, 95% *CI* 1.05 to 2.15). Emotional abuse (*OR* = 1.88, 95% *CI* 1.18 to 3.02) and neglect (*OR* = 1.45, 95% *CI* 1.01 to 2.09) were positively associated with the suicide attempts class. Further details are presented in **Table 4**.

**Table 4:** Associations between ACEs types and three classes of NSSI and SBs.

ACE types	Suicidal ideation class			Suicide attempts class		
	n (%)	*aOR* ^#^ (95% *CI*)	*aOR* ^&^ (95% *CI*)	n (%)	*aOR* ^#^ (95% *CI*)	*aOR* ^&^ (95% *CI*)
Physical abuse						
Yes	105 (39.2)	1.54 (1.11~2.14) ^*^	1.50 (1.05 ~ 2.15) ^*^	40 (14.9)	1.49 (0.90~2.47)	1.46 (0.84 ~ 2.51)
No	1061 (22.1)	1.00	1.00	181 (3.8)	1.00	1.00
Emotional abuse						
Yes	157 (47.4)	3.02 (2.24~4.08) ^***^	1.27 (0.90 ~ 1.78)	77 (23.3)	7.14 (4.71~10.81) ^***^	1.88 (1.18 ~ 3.02) ^**^
No	1009 (21.4)	1.00	1.00	144 (3.1)	1.00	1.00
Sexual abuse						
Yes	62 (29.8)	0.96 (0.67~1.38)	0.70 (0.48 ~ 1.03)	28 (13.5)	1.73 (1.02~2.93) ^*^	1.23 (0.70 ~ 2.17)
No	1104 (22.8)	1.00	1.00	193 (4.0)	1.00	1.00
Neglect						
Yes	270 (35.4)	1.72 (1.41~2.09) ^***^	1.10 (0.89 ~ 1.37)	88 (11.5)	2.46 (1.75~3.46) ^***^	1.45 (1.01 ~ 2.09) ^*^
No	896 (20.9)	1.00	1.00	133 (3.1)	1.00	1.00
Household substance abuse						
Yes	119 (35.2)	1.47 (1.12~1.94) ^**^	1.22 (0.91 ~ 1.65)	36 (10.7)	1.72 (1.08~2.74) ^*^	1.43(0.87 ~ 2.35)
No	1047 (22.3)	1.00	1.00	185 (3.9)	1.00	1.00
Parental separation/divorce						
Yes	345 (29.8)	1.34 (1.13~1.59) ^***^	1.15 (0.96 ~ 1.39)	85 (7.3)	1.62 (1.17~2.24) ^**^	1.27 (0.90 ~ 1.80)
No	821 (21.1)	1.00	1.00	136 (3.5)	1.00	1.00
Witnessed mother abused						
Yes	89 (36.8)	1.40 (1.11~1.76) ^**^	1.22 (0.95 ~ 1.56)	55 (10.7)	1.55 (1.04~2.30) ^*^	1.35 (0.88 ~ 2.05)
No	977 (21.6)	1.00	1.00	166 (3.7)	1.00	1.00
Household mental illness						
Yes	55 (34.4)	1.18 (0.79~1.75)	0.98 (0.64 ~ 1.50)	24 (15.0)	1.85 (1.04~3.30) ^*^	1.53 (0.82 ~ 2.86)
No	1111 (22.8)	1.00	1.00	197 (4.0)	1.00	1.00
Household incarceration						
Yes	44 (34.4)	1.24 (0.81~1.91)	1.22 (0.76 ~ 1.94)	10 (7.8)	0.94 (0.43~2.08)	0.81 (0.34 ~ 1.92)
No	1122 (22.8)	1.00	1.00	211 (4.3)	1.00	1.00

*aOR*, adjusted odds ratio; ACE, adverse childhood experiences; ^#^Adjusted grade, gender, boarding status, family economic status, academic performance, learning burden, number of intimate friends, and other 8 types of ACEs. ^&^Add depression scores on the basis of ^#^. Bold values indicate statistically significant results. ^*^p < 0.05, ^**^p < 0.01, ^***^p < 0.001.

### 3.5 Gender differences in the associations between ACE types and the co-occurrence patterns

**Figure 2:**
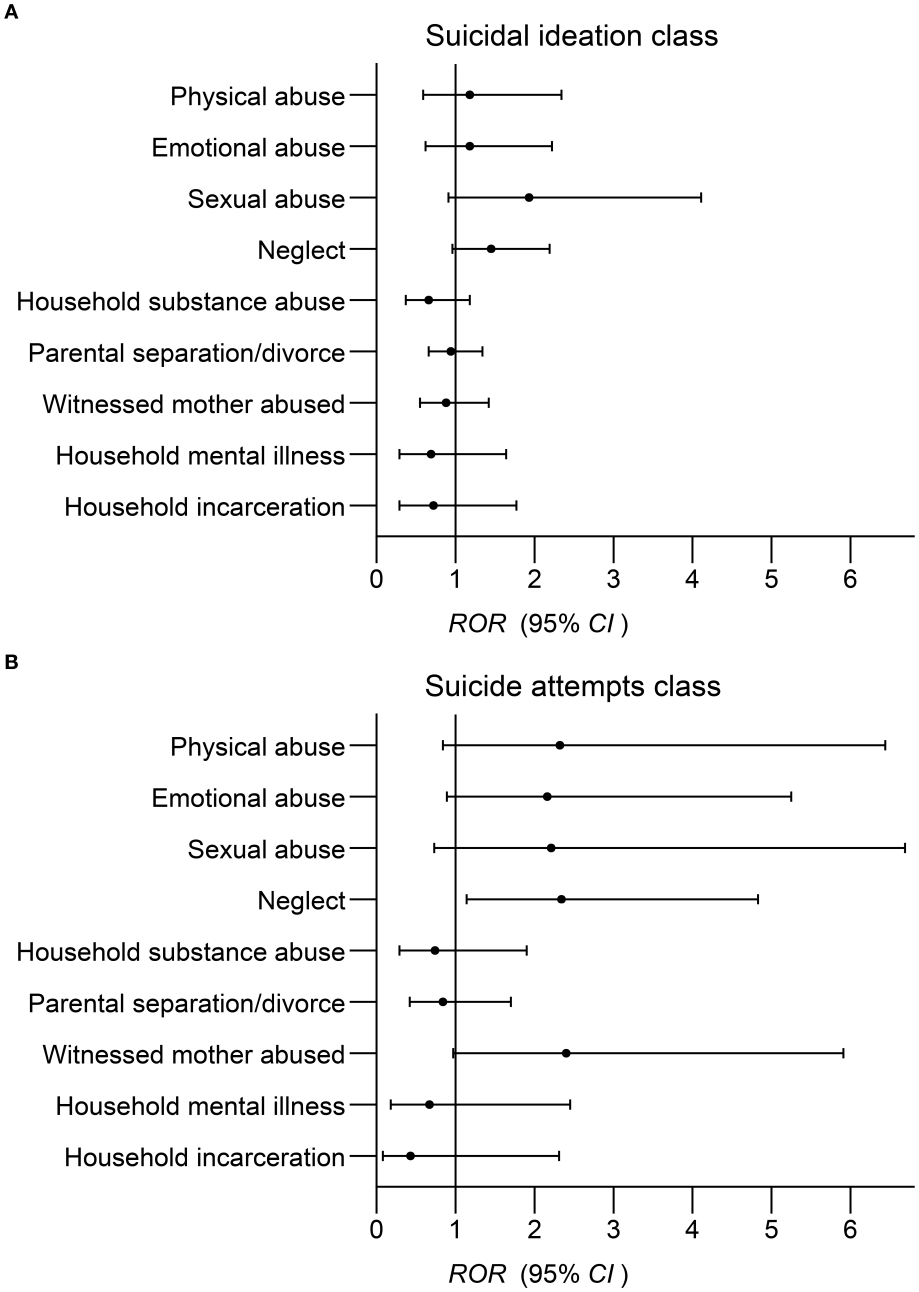
**(A)** Gender differences in the associations between ACEs types and suicidal ideation class; Relative odds ratios (*ROR*) in females vs. males. Adjusted grade, gender, boarding status, family economic status, academic performance, learning burden, number of intimate friends, depression scores, and other 8 types of ACEs. **(B)** Gender differences in the associations between ACEs types and suicide attempts class; Relative odds ratios (*ROR*) in females vs. males. Adjusted grade, gender, boarding status, family economic status, academic performance, learning burden, number of intimate friends, depression scores, and other 8 types of ACEs.

Compared with low risk class, no statistically significant gender differences were found in the associations between any types of ACEs and suicidal ideation class (*ps* all > 0.05). However, females exposed to neglect were more likely to engage in suicide attempts class than males (*ROR* = 2.34, 95% *CI* 1.14 to 4.83), with this difference being statistically significant (*p* < 0.05). The results are illustrated in **Figure 2**.

## 4 Discussion

This is the first study to examine the exact effects of different ACE types on the co-occurrence of NSSI and three stages of SBs among adolescents, as well as the potential moderating role of gender. Our study yields several major findings. First, three classes were identified via LCA, with participants showing a tendency to experience NSSI and SBs concurrently rather than in isolation. Notably, individuals with SA and SI are at a higher risk of co-occurring NSSI. Second, distinct ACE types have non-equivalent effects on the co-occurrence patterns. In particular, physical abuse is the strongest risk factor for the co-occurrence of NSSI and SI (NSSI + SI), while emotional abuse and neglect are identified as robust risk factors for the co-occurrence of NSSI and SA (NSSI + SA). Third, the impacts of ACE types on the co-occurrence patterns differ by gender. Specifically, female adolescents exposed to neglect are more likely to develop NSSI + SA. These findings enhance our understanding of the relationship between NSSI and SBs in adolescents. They may also assist policy-makers in developing targeted prevention and intervention strategies for co-occurring self-harm behaviors.

The results of the LCA in this study are similar to those of a previous study (21), showing alignment in both methodology and outcomes. The proportion of suicidal ideation class is comparable (23.1% vs.25.0%), while the suicide attempts class is substantially lower in our study (4.4% vs. 29.9%). This marked discrepancy is primarily due to notable methodological differences, including variations in study populations, measurement instruments, and operational definitions. In contrast, the proportion of suicide attempts class in our study is close to the prevalence of NSSI + SA reported in a meta-analysis of Chinese adolescents (16), providing better population-specific comparability. However, the meta-analysis did not adopt LCA for classification, making direct comparison of behavioral co-occurrence patterns difficult. A study conducted among South Korean adults, which used the same variables (NSSI, SI, SP, and SA) in its LCA, also identified three subgroups (63). This study provides the closest comparability to our findings in terms of methodology and outcomes. The feature of the suicidal ideation class in our study differs from the second subgroup of the South Korean study, which was characterized by high SI risk and low probability of other behaviors. In contrast, the suicide attempts class in our study shares similar co-occurrence features with the first subgroup, though the proportion is larger in our sample (4.4% vs. 1.8%). This suggests that adolescents are more likely to experience NSSI and SBs concurrently. NSSI + SI is unique and common among adolescents, and NSSI + SA is more prevalent in this group.

The results of other studies are not directly comparable to those of the present study (19, 20, 23, 64). First, the variables included in the LCA vary from those in our study. For example, a study on Chinese adolescents classified the participants into four classes based on 11 types of self-injurious behaviors (20), whereas in that study, NSSI behaviors were concrete manifestations, such as self-cutting and dangerous drinking. Second, the populations targeted by these studies also differ. Herres et al. focused on pediatric emergency department patients (64), while Bryan et al. examined gun owners (23).

In addition, our study found that NSSI and SBs tend to co-occur, which aligns with prior research (14, 15, 44, 65). However, most of these studies have adopted a variable-centered perspective. Adolescents with SA and SI are at a higher risk of experiencing co-occurring NSSI, which may seem paradoxical, as the definition of NSSI explicitly excludes suicidal thoughts. To explain the relationship between NSSI and suicide, several theoretical models have been proposed. The Anti-Suicide Model views NSSI as an active coping mechanism used to avoid suicide, with individuals channeling destructive impulses into NSSI to prevent complete self-destruction (66). The Gateway Theory posits that NSSI and suicide exist along a continuum of self-injury behaviors, with NSSI at one extreme and suicide death at the other (37, 67). Recently, the Interpersonal Theory of Suicide has gained increasing popularity, which emphasizes that individuals who wish to commit suicide must possess the capability to do so and overcome the fear of death (16, 39). A key distinction between the latter two theories is that the former suggests that NSSI is a necessary precursor to suicide, whereas the latter does not (37). Our study indicates that not all adolescents who engage in NSSI exhibit this behavior without genuine suicidal thoughts. This may be due to the fact that adolescents’ cognitive development is still maturing, making it difficult for them to accurately distinguish whether their self-harm is intended to end their lives. Alternatively, suicidal thoughts could be latent or unconscious, with the individuals themselves unaware of underlying feelings. Overall, our findings emphasize the need to consider the co-occurrence of both behaviors in future research and practice.

Our results demonstrate that the effects of different ACE types on the co-occurrence patterns of NSSI and SBs vary in adolescents. For one thing, the mechanisms underlying these effects may differ across ACE types (68, 69). For example, Peng et al.’s study suggests that the impact of sexual abuse on suicide risk is usually acute (54). For another, the effects of ACEs may be influenced by factors such as timing, frequency (severity), and duration, none of which were taken into account in the design of this study (68, 70). Similar conclusions have been drawn in other studies, highlighting that different ACE types have non-equivalent contributions to both NSSI and SBs (31, 32). Some prior studies have treated ACE types as equal by summing them to create a total ACE score (51, 71). Our research indicates that distinct types of ACEs should be assigned different weights when exploring their impacts on NSSI + SBs. Furthermore, our findings show that physical abuse is the strongest risk factor for NSSI + SI, while emotional abuse and neglect appear more strongly associated with NSSI + SA. According to Joiner’s Interpersonal Theory of Suicide (39), a pathway from SI to NSSI and then to SA is plausible. Our study suggests that physical abuse may primarily facilitate the progression from SI to NSSI, whereas emotional abuse and neglect seem to have a more significant role in advancing NSSI into SA, a more severe outcome. Overall, these findings provide a valuable reference for the development of precise and targeted interventions for addressing the co-occurrence pattern of self-injury behavior among adolescents.

Our study found that female adolescents were more likely to experience co-occurring NSSI and SBs, which is consistent with previous findings (36, 45). This may be partly explained by the gender differences in emotional traits. Girls tend to be more sensitive to emotional attitudes and related deficiencies (72). In addition, we observed gender differences in the prevalence of specific ACE types, consistent with other studies (50, 73). In our sample, boys were more likely to experience physical abuse, whereas girls were more likely to experience emotional abuse. This aligns with the traditional Chinese concept of “a dutiful son grows up under the rod” and the societal perception that girls are more physically vulnerable, as these factors may lead caregivers to impose physical violence more often on boys and verbal/emotional attacks more often on girls. Notably, the prevalence of sexual abuse was higher among boys, which is consistent with previous research (74). However, this may not fully reflect reality, as Chinese girls may be less likely to disclose sexual abuse due to feelings of shame and the influence of traditional cultural norms.

More importantly, the present findings reveal notable gender differences in the associations between specific ACE types and co-occurrence patterns of NSSI and SBs. In particular, neglect exerted a stronger effect on NSSI + SA among girls. This moderating role of gender has also been reported in a prior study examining the effect of ACEs on suicide risk (54). According to the theory of gender role orientation, individuals tend to internalize gender-typed characteristics that align with their sociocultural context. Such an identification process may, in turn, shape how ACEs influence subsequent behavioral and psychological outcomes (74).

Our study provides valuable implications for public health practice. First, NSSI and SBs tend to co-occur among adolescents, indicating that health professionals should conceptualize these behaviors jointly and implement integrated intervention strategies targeting both outcomes. Second, physical abuse, emotional abuse, and neglect are strong risk factors for NSSI + SBs. This suggests that prevention and intervention efforts for the co-occurrence patterns should prioritize these specific ACE types. Strengthening policies to reduce child maltreatment, alongside community-based programs that promote positive parenting and effective emotional communication, may help mitigate these risks. Finally, gender-specific approaches are warranted. Public health practitioners should be particularly attentive to the heightened impact of neglect on female adolescents when designing and delivering interventions.

## 5 Limitations

There are still some limitations in this study. First, the cross-sectional design restricts our ability to draw causal inferences regarding the associations between ACEs and NSSI + SBs. Future studies could employ longitudinal designs to strengthen causal interpretation. Second, the representativeness of our sample is limited. Data were collected exclusively in Chongqing, and only junior high school students were included. Multi-center studies with more diverse samples, including senior high school students, are needed to improve generalizability. Third, the use of a self-report questionnaire may introduce the reporting and recall biases, particularly in measuring ACEs. Some ACE types, such as sexual abuse and household incarceration, involve sensitive personal experiences that may be underreported. Future studies should incorporate multiple informants (such as adolescents, guardians, and teachers) and use both retrospective and prospective assessments to improve measurement accuracy. Fourth, the current study did not distinguish between NSSI thoughts and NSSI behaviors. Subsequent research could adopt the self-injurious thoughts and behaviors (SITBs) framework (21) to achieve a more comprehensive understanding. Additionally, although the present study included a wide range of ACE types, it did not cover all possible adversity items. Future research could utilize the Adverse Childhood Experiences-International Questionnaire (ACE-IQ) recommended by the WHO to conduct a more comprehensive assessment (25). It is also worth noting that dichotomizing Childhood Trauma Questionnaire (CTQ) scores inevitably leads to information loss. Future studies are encouraged to retain the original continuous CTQ scores. Finally, this study focused on identifying NSSI + SBs and their associations with ACEs, but did not investigate potential mediating mechanisms (e.g., depression symptoms). Future research should incorporate mediation analyses to systematically explore these underlying pathways.

## 6 Conclusions

This study examined the unique effects of specific ACE types on NSSI + SBs among Chinese adolescents. Three co-occurrence patterns of NSSI and SBs were identified among adolescents, specifically that SA and SI commonly co-occur alongside NSSI. Different ACE types exerted non-equivalent influences on these patterns, and gender difference was also observed. These findings underscore the importance of considering NSSI and SBs simultaneously, and it is more applicable and promising to develop tailored, ACEs-specific prevention and intervention strategies.

## Data Availability

The raw data supporting the conclusions of this article will be made available by the authors, without undue reservation.
