# Correction: Specific effects of distinct types of adverse childhood experiences on the co-occurrence of non-suicidal self-injury and suicidal behaviors among Chinese adolescents: a latent class analysis

**DOI:** 10.3389/fpsyt.2026.1857626

**Published:** 2026-04-23

**Authors:** Frontiers Production Office

**Affiliations:** Frontiers Media SA, Lausanne, Switzerland

**Keywords:** adolescent, adverse childhood experiences, latent class analysis, non-suicidal self-injury, suicidal behaviors

The citation for this article was erroneously given as “Wang Z, Yang S, Chen W, Chen G, Wan X, Li X, Peng C and Wang H (2026) Specific effects of distinct types of adverse childhood experiences on the co-occurrence of non-suicidal self-injury and suicidal behaviors among Chinese adolescents: a latent class analysis. *Front. Psychiatry* 16:1698537. doi: 10.3389/fpsyt.2025.1698537”. This does not match the published author list, which reads: “Zhouyan Wang^1,2^, Wei Chen^1,2,3^, Siwei Yang^1,2^, Gen Chen^1,2^, Xiaoke Wan^1,2^, Xia Li^1,2,4^, Chang Peng^1,2*†^ and Hong Wang^1,2*†^” (with Wei Chen listed before Siwei Yang). The correct citation is:

“Wang Z, Chen W, Yang S, Chen G, Wan X, Li X, Peng C and Wang H (2026) Specific effects of distinct types of adverse childhood experiences on the co-occurrence of non-suicidal self-injury and suicidal behaviors among Chinese adolescents: a latent class analysis. *Front. Psychiatry* 16:1698537. doi: 10.3389/fpsyt.2025.1698537”

The original version of this article has been updated.

