# Supplementary material for: Specific effects of distinct types of adverse childhood experiences on the co-occurrence of non-suicidal self-injury and suicidal behaviors among Chinese adolescents: a latent class analysis
Source: Front Psychiatry. 2026 Jan 12;16:1698537. doi: 10.3389/fpsyt.2025.1698537 (PMC12832740; doi:10.3389/fpsyt.2025.1698537)
Supplement: Supplementary file 1 [file DataSheet1.docx]

**Table S1**

Fit indices for latent class models of NSSI and SBs (Males).

| Model | AIC | BIC | aBIC | Entropy | LMR | BLRT | n (%) | | | | |
| --- | --- | --- | --- | --- | --- | --- | --- | --- | --- | --- | --- |
|  |  |  |  |  | *P* | *p* | 1 | 2 | 3 | 4 | 5 |
| 1 | 6515.493 | 6538.715 | 6526.006 | 1.000 |  |  | 2,454 (100.00) |  |  |  |  |
| 2 | 5169.541 | 5221.790 | 5193.195 | 0.917 | <0.001 | <0.001 | 2,156 (87.86) | 298 (12.14) |  |  |  |
| **3** | **5095.031** | **5176.308** | **5131.826** | **0.907** | **<0.001** | **<0.001** | **204 (8.31)** | **317 (12.92)** | **1,933 (78.77)** |  |  |
| 4 | 5105.031 | 5215.335 | 5154.968 | 0.694 | 0.4998 | 1.0000 | 0 (0.00) | 1,933 (78.77) | 73 (2.98) | 448 (18.26) |  |
| 5 | 5115.031 | 5254.363 | 5178.109 | 0.598 | 0.5011 | 1.0000 | 204 (8.31) | 0 (0.00) | 0 (0.00) | 1,813 (73.88) | 437 (17.81) |

AIC: Akaike Information Criterion; BIC: Bayesian Information Criterion; aBIC: sample-size adjusted BIC; LMR: Lo-Mendell-Rubin adjusted likelihood ratio test; BLRT: Bootstrap Likelihood Ratio Test.

**Table S2**

Fit indices for latent class models of NSSI and SBs (Females).

| Model | AIC | BIC | aBIC | Entropy | LMR | BLRT |  |  | n (%) |  |  |
| --- | --- | --- | --- | --- | --- | --- | --- | --- | --- | --- | --- |
|  |  |  |  |  | *p* | *p* | 1 | 2 | 3 | 4 | 5 |
| 1 | 9243.651 | 9267.082 | 9254.373 | 1.000 |  |  | 2,586 (100.00) |  |  |  |  |
| 2 | 7164.014 | 7216.735 | 7188.140 | 0.884 | <0.001 | <0.001 | 556 (21.50) | 2,030 (78.50) |  |  |  |
| **3** | **6990.710** | **7072.720** | **7028.238** | **0.877** | **<0.001** | **<0.001** | **718 (27.77)** | **1,720 (66.51)** | **148 (5.72)** |  |  |
| 4 | 7000.710 | 7112.009 | 7051.641 | 0.723 | 0.5017 | 1.0000 | 0 (0.00) | 1,720 (66.51) | 148 (5.72) | 718 (27.77) |  |
| 5 | 7010.710 | 7151.298 | 7075.044 | 0.7074 | 1.0000 | 1.0000 | 273 (10.56) | 111 (4.29) | 428 (16.55) | 1,602 (61.95) | 172 (6.65) |

AIC: Akaike Information Criterion; BIC: Bayesian Information Criterion; aBIC: sample-size adjusted BIC; LMR: Lo-Mendell-Rubin adjusted likelihood ratio test; BLRT: Bootstrap Likelihood Ratio Test.

**Table S3**

Item-response probabilities for the three latent classes of NSSI and SBs (Total).

| Variables | Category | Class 1 | Class 2 | Class 3 |
| --- | --- | --- | --- | --- |
| NSSI | 0 | 0.935 (0.927-0.943) | 0.583 (0.555-0.612) | 0.276 (0.217-0.335) |
|  | 1 | **0.065 (0.057-0.073)** | **0.417 (0.388-0.45)** | **0.724 (0.665-0.783)** |
| SI | 0 | 0.950 (0.936-0.965) | 0.000 (0.000-0.000) | 0.000 (0.000-0.000) |
|  | 1 | **0.050 (0.035-0.064)** | **1.000 (1.000-1.000)** | **1.000 (1.000-1.000)** |
| SP | 0 | 1.000 (1.000-1.000) | 0.715 (0.675-0.754) | 0.000 (0.000-0.000) |
|  | 1 | **0.000 (0.000-0.000)** | **0.285 (0.246-0.325)** | **1.000 (1.000-1.000)** |
| SA | 0 | 1.000 (1.000-1.000) | 1.000 (1.000-1.000) | 0.359 (0.253-0.465) |
|  | 1 | **0.000 (0.000-0.000)** | **0.000 (0.000-0.000)** | **0.641 (0.535-0.747)** |

NSSI: non-suicidal self-injury; SI: suicidal ideation; SP: suicide plans; SA: suicide attempts.

**Table S4**

Item-response probabilities for the three latent classes of NSSI and SBs (Males).

| Variables | Category | Class 1 | Class 2 | Class 3 |
| --- | --- | --- | --- | --- |
| NSSI | 0 | 0.425 (0.311-0.538) | 0.616 (0.439-0.793) | 0.938 (0.927-0.949) |
|  | 1 | **0.575 (0.462-0.689)** | **0.384 (0.207-0.561)** | **0.062 (0.051-0.073)** |
| SI | 0 | 0.000 (0.000-0.000) | 0.000 (0.000-0.000) | 0.957 (0.905-1.010) |
|  | 1 | **1.000 (1.000-1.000)** | **1.000 (1.000-1.000)** | **0.043 (-0.010-0.095)** |
| SP | 0 | 0.000 (0.000-0.000) | 0.825 (0.421-1.229) | 1.000 **(1.000-1.000)** |
|  | 1 | **1.000 (1.000-1.000)** | **0.175 (-0.229-0.579)** | **0.000** (0.000-0.000) |
| SA | 0 | 0.529 (0.174-0.884) | 1.000 (1.000-1.000) | 1.000 **(1.000-1.000)** |
|  | 1 | **0.471 (0.116-0.826)** | **0.000** (0.000-0.000) | **0.000** (0.000-0.000) |

NSSI: non-suicidal self-injury; SI: suicidal ideation; SP: suicide plans; SA: suicide attempts.

**Table S5**

Item-response probabilities for the three latent classes of NSSI and SBs (Females).

| Variables | Category | Class 1 | Class 2 | Class 3 |
| --- | --- | --- | --- | --- |
| NSSI | 0 | 0.527 (0.448-0.605) | 0.931 (0.919-0.943) | 0.203 (0.138-0.267) |
|  | 1 | **0.473 (0.395-0.552)** | **0.069 (0.057-0.081)** | **0.797 (0.733-0.862)** |
| SI | 0 | 0.000 (0.000-0.000) | 0.925 (0.885-0.966) | 0.000 (0.000-0.000) |
|  | 1 | **1.000 (1.000-1.000)** | **0.075 (0.034-0.115)** | **1.000 (1.000-1.000)** |
| SP | 0 | 0.638 (0.561-0.715) | 1.000 **(1.000-1.000)** | 0.000 (0.000-0.000) |
|  | 1 | **0.362 (0.285-0.439)** | **0.000** (0.000-0.000) | **1.000 (1.000-1.000)** |
| SA | 0 | 1.000 **(1.000-1.000)** | 1.000 **(1.000-1.000)** | 0.218 (0.096-0.339) |
|  | 1 | **0.000** (0.000-0.000) | **0.000** (0.000-0.000) | **0.782 (0.661-0.904)** |

NSSI: non-suicidal self-injury; SI: suicidal ideation; SP: suicide plans; SA: suicide attempts.


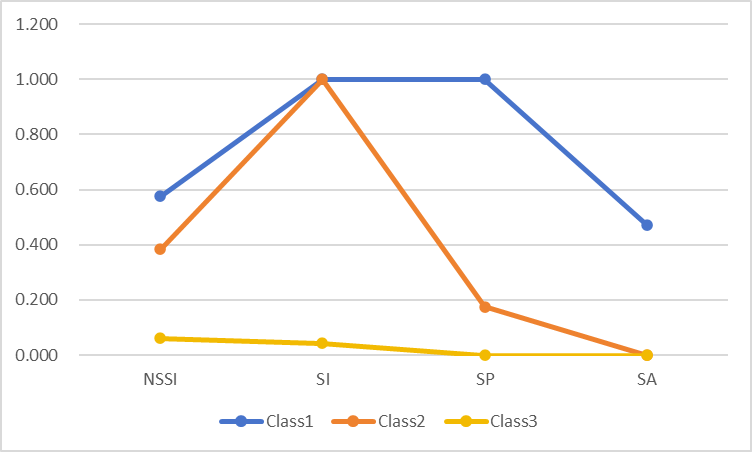


**Figure S1.** Plots of three latent classes for the NSSI and SBs (Males).

NSSI: non-suicidal self-injury; SI: suicidal ideation; SP: suicide plans; SA: suicide attempts.


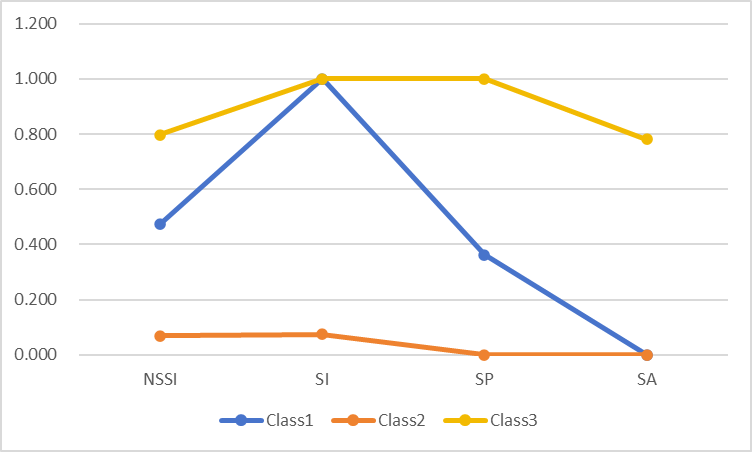


**Figure S2. Plots of three latent classes for the NSSI and SBs (Females).**

NSSI: non-suicidal self-injury; SI: suicidal ideation; SP: suicide plans; SA: suicide attempts.
